# A feasibility study to assess *Imbrasia belina* (mopane worm) sensitisation and related respiratory health outcomes in a rural community in Gwanda district, Zimbabwe

**DOI:** 10.1186/s40814-021-00780-9

**Published:** 2021-02-22

**Authors:** Vuyelwa Ndlovu, Moses Chimbari, Elopy Sibanda, Pisirai Ndarukwa

**Affiliations:** 1 School of Nursing and Public Health, College of Health Sciences, Howard College Campus, University of KwaZulu-Natal, Durban, South Africa; 2 Department of Environmental Science and Health, Faculty of Applied Sciences, National University of Science and Technology, Corner Gwanda Road and Cecil Avenue, PO Box AC 939, Ascot, Bulawayo, Zimbabwe; 3 Asthma, Allergy and Immune Dysfunction Clinic, Twin Palms Medical Centre, 113 Kwame Nkrumah Avenue, Harare, Zimbabwe; 4 Department of Pathology, Medical School, National University of Science and Technology, Bulawayo, Zimbabwe

**Keywords:** Asthma, Spirometry, FeNO, Allergy, Mopane worm sensitisation, Edible insects, Feasibility study

## Abstract

**Background:**

Allergic diseases are considered to be some of the fastest growing chronic conditions in Africa. Of concern is the paucity of knowledge about the local environment and its role in allergic disease development. In response to this, we explored whether *Imbrasia belina*, a popular indigenous edible insect commonly known as mopane worm, is a potential allergen of clinical and public health significance in Zimbabwe. This study was intended to assess the plausibility and feasibility of this hypothesis with a view to evaluate the insect’s health impact in a larger study.

**Methods:**

The study participants included male and female villagers aged 10 years and above in Gwanda district, Zimbabwe. Eligible participants who completed the household questionnaire were referred to the local clinic for skin prick tests and to measure lung function and allergic airway inflammation. Allergen sensitisation patterns were evaluated using 10 different inhalant allergen extracts including an in-house preparation of mopane worm. Lung function was measured with a Koko Legend spirometer, and fractional exhaled nitric oxide levels (FeNO) (NIOX VERO) were measured in participants with at least one abnormal spirometric parameter. Data was analysed using Stata version 13 software.

**Results:**

Of the 46 eligible participants that completed the household questionnaire, 17 went to the clinic giving a response rate of 37%. The majority who completed the questionnaire were adults (91%) and the children (9%) were all female. The prevalence of sensitisation to *Imbrasia belina* was 50%, and the prevalence ranged from 22 to 72% for the other allergens including cockroach, mosquito and house dust mites. The data collection tools were safe and well tolerated by participants with no adverse events reported. Self-reported respiratory symptoms, abnormal lung function and elevated FeNO were recorded amongst participants sensitised to mopane worm.

**Conclusion:**

Pre-defined feasibility criteria were met with the exception of a lower than expected response rate for clinic data collection in this pilot study. For the main study, modifying the sampling strategy and applying more consistent community engagement will improve the response rates.

**Supplementary Information:**

The online version contains supplementary material available at 10.1186/s40814-021-00780-9.

## Key messages regarding feasibility

- What uncertainties existed regarding the feasibility?
  It is unknown whether *Imbrasia belina*, a popular indigenous edible insect commonly known as mopane worm, is potentially an allergen of clinical and public health significance in Zimbabwe. An assessment of the feasibility of carrying out a study of this nature in remote settings was required.
- What are the key feasibility findings?
  Of the 46 eligible participants that completed the household questionnaire, 17 went to the clinic giving a response rate of 37%. There were more adults (91%) than children (9%) participating. The prevalence of sensitisation to *Imbrasia belina* was 50%. The data collection tools were safe and well tolerated by participants. Respiratory health symptoms were recorded amongst participants sensitised to mopane worm.
- What are the implications of the feasibility findings for the design of the main study?
  Feasibility criteria were met with the exception of a low response rate for clinic data collection. Strategies will be put in place to improve the response rates.

## Background

There is a paucity of published epidemiological information about allergic diseases in Africa despite the rapidly increasing burden [1]. This is largely due to limited funding and expertise in allergy research [2] as well as the prioritisation of infectious diseases such as HIV/AIDS, tuberculosis, malaria and recently COVID-19. Much of the research in the field of allergy is currently conducted in high-income countries [3–5], and generalisability of such findings to African countries is challenging due to differences in genetic, environmental and lifestyle characteristics. The allergy profile of African patients is influenced by the flora and fauna that locals depend on for food and other purposes [6]. The exposure to parasitic infections from early childhood [5, 7–9] and uncontrolled or unregulated exposure to many irritant pollutants such as dust, smoke and pesticides [4, 10] presents unique circumstances warranting investigations that are pivoted from an African perspective.

The rapid emergence of asthma and other allergic diseases in the African continent is believed to be a result of ongoing lifestyle and dietary changes that are occurring in the midst of climate change, economic development and westernisation [2]. The exact nature and extent of these environmental influences is still largely unexplored especially considering their interaction with the vast genomic and ethnic diversity existing in the African population. Furthermore, there are many local exposures whose role as important allergens is yet to be investigated and documented [11]. To make progress towards understanding the allergy epidemic in Africa requires careful consideration of the contextually relevant risk factors.

There is currently limited asthma and allergy research in Zimbabwe compared to other African countries [12]. The few studies that have been done, however, suggest that prevalence may be increasing [13, 14]. The strong reliance on natural resources for livelihoods in Zimbabwe [5, 7, 15] is an indicator that there is a possible presence of allergic sensitisation to some of the local environmental exposures. Entomophagy, a common practice in Zimbabwe, is a recognised source of neo-allergens occurring through occupational exposure or ingestion [16]. *Imbrasia belina*, a popular indigenous edible insect, commonly known as ‘mopane worm’ has already been documented to be a clinically relevant allergen source in Botswana and Zimbabwe [17, 18]. Furthermore, sensitisation to other edible insects has been found to be associated with asthma and respiratory allergy [16] particularly amongst those occupationally exposed [19–21]. It is estimated that income from mopane worm harvesting may contribute up to a quarter of total annual cash income for rural households [22]. As a result of this shift from harvesting for subsistence to commercial purposes, exposure to the worm has increased.

It is in response to this logic that we considered exploring whether mopane worm could potentially be an allergen of clinical and public health significance in Zimbabwe, particularly in communities where exposure is very high. A mixed methods research study entitled the Gwanda Asthma and Respiratory Allergy Study (GARAS) was thus designed to test this hypothesis. A study addressing entomophagy and respiratory allergy in a rural community setting has not been conducted in Zimbabwe. This will be the first extensive study investigating *Imbrasia belina* as an allergen of clinical significance in a vulnerable community frequently exposed to it. This feasibility study was conducted with the main aim of assessing the possibility of carrying out a study of this nature in a rural community in the Gwanda district of Zimbabwe. More specifically the study sought to assess the influence of the study’s proposed recruitment strategy on the response rate. Secondly, to identify challenges in the collection of information using a household questionnaire. The third objective was to evaluate the safety and acceptability of the clinic data collection tools and procedures in a rural clinic. Because evidence of mopane worm allergy is found only in a few case reports, the fourth objective was to explore the plausibility of population-wide sensitisation and its clinical relevance in the study area. The last objective was to evaluate the relevance of the proposed panel of allergens in the study area.

## Methods

### Study design and setting

**Fig. 1:**
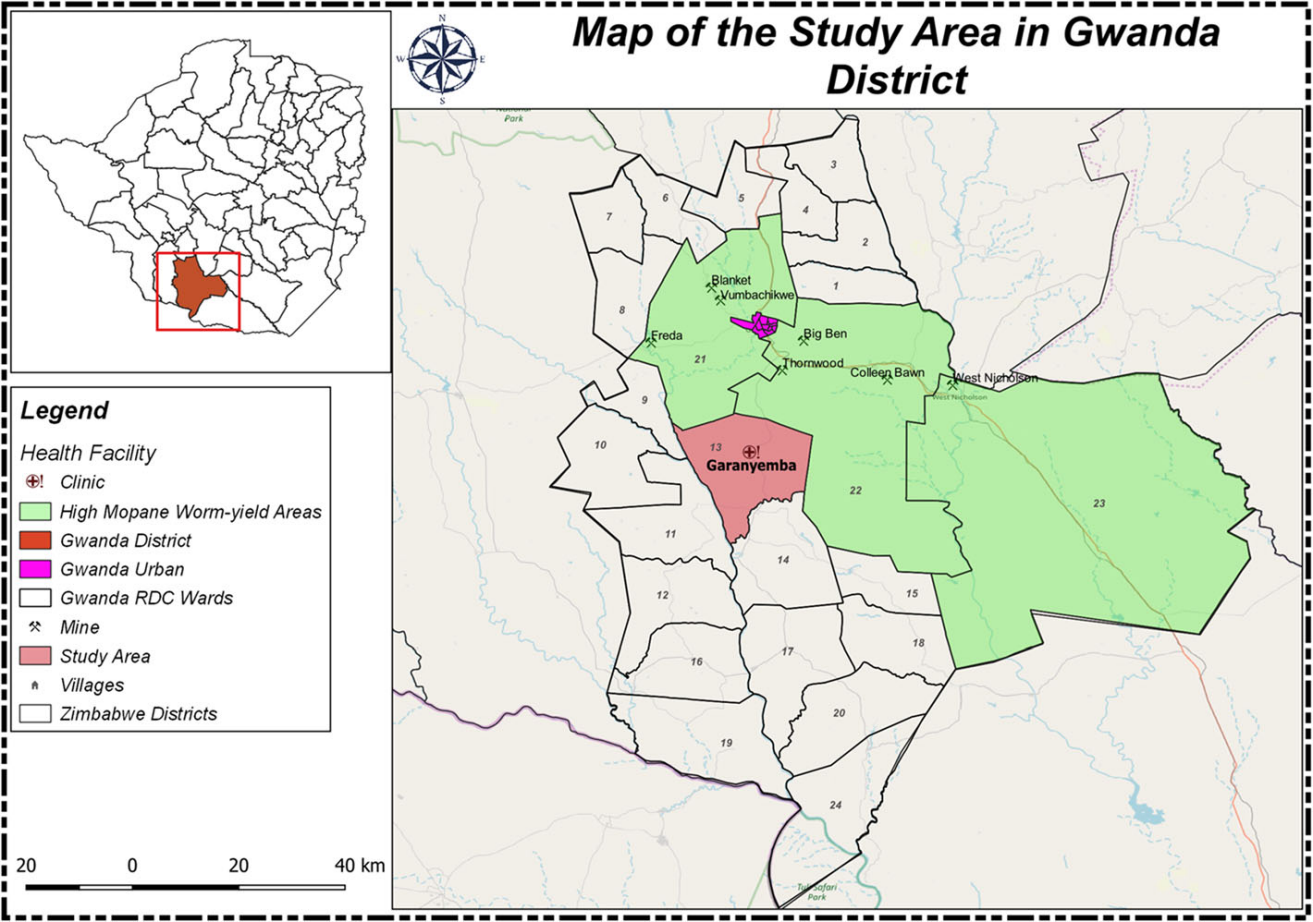
Map of the study area in Gwanda district, Matabeleland South Province in Zimbabwe

This cross-sectional study was conducted in Gwanda district, one of Zimbabwe’s main sources of *Imbrasia belina*. Gwanda district, presented in Fig. 1, lies in Matabeleland South province. In Zimbabwe, provinces are divided into districts, districts are further divided into smaller administrative units called wards and the wards are subdivided into villages.

Prior to the feasibility study, a series of community engagement (CE) strategies were implemented from the 14th of May to the 30th of October 2019 with the aim of securing support and ensuring that this research project is aligned to the sociocultural, political and economic contexts in Gwanda. The CE activities utilised in this study had been successfully carried out in a previous study in the area and had included advisory meetings with local leaders, workshops and research training for local youth [23]. One of the most important CE activities carried out in this study was the consultation of community leaders and other stakeholders to assist the research team in identifying the ideal study site in the district. Four main study site requirements were taken into consideration. The first requirement was the availability of a centrally located and fully functional clinic that could accommodate our data collection equipment. The second requirement was good access to the main road and Gwanda town, where the hospital is located, in the event of an emergency. The third requirement was availability and proximity of eligible participants. Finally, the fourth requirement was ubiquitous exposure to mopane worm. While all the wards in the district were reported to produce mopane worm, 3 wards were reported by various stakeholders to be producers of some of the highest yields of mopane worm in Gwanda district probably due to low human population density [24] and an abundance of *Colophospermum mopane* tree species which the mopane worm primarily feeds on [25]. The rough terrain and widely scattered dwellings in these wards, though good for the proliferation of mopane worm, were not ideal for this study. As illustrated in Fig. 1, Garanyemba was selected as the study site because it met all the 4 requirements. It is a communal settlement with 8 villages, a total population of 7811, 1643 households and an average household size of 4.8 [25]. The ward also has good roads and a centrally located, fully functional clinic which was ideal for the clinical assessments. In addition to mopane worm harvesting around the homesteads, Garanyemba’s close proximity to *Imbrasia belina* hotspots [24] means villagers are able to travel to these sites and they often establish temporary shelter there for days or weeks to harvest as much as possible [26]. The inclusion criteria were males and females aged 10 years and over residing in Garanyemba, Gwanda district, for at least a year. Previous surveys in Zimbabwe and Botswana reported that children as young as 10 years old participate in mopane worm harvesting in various ways [26]. Children and adults not residing in Gwanda for at least a year were excluded. The study was approved by the Medical Research Council of Zimbabwe (Ref number MRCZ/A/2486) and the University of KwaZulu-Natal’s Biomedical Research Ethics Committee BREC (Ref number BE 327/19).

### Sample size and feasibility criteria

Due to budgetary limitations, cost was also a significant consideration for feasibility assessment. The main study had to be accommodated within the available budget; therefore, approximate anticipated costs per day during fieldwork were calculated. To efficiently utilise the available financial resources, we determined that data would have to be collected from 10% of the total sample per day during the main study.

For the main study, a total sample size of 462 was calculated using Cochran’s formula [27] based on the assumption of a prevalence estimate of 50% for mopane worm sensitisation, a 5% margin of error at 95% confidence interval and adjusted for anticipated non-response (20%). It was planned that the 462 participants would then be recruited from all the 8 villages using systematic random sampling with allocation of the target number of households in each village being proportional to size.

We therefore intended to recruit 46 eligible participants in order to evaluate the feasibility of the study procedures over a 2-day period in the context of the available funds. The first day was allocated for the household questionnaire and the second day was for clinic data collection. Having approached the participants in their households to complete the questionnaire on day 1, it was not expected that they would be readily available, on such short notice, to go to the clinic on the same day. The decision to collect clinic data on the second day was a logistical one but it was also an opportunity for the participants to reflect on the study information provided and make an informed decision. It is on this basis and in line with other objectives for this pilot study that feasibility criteria for success were developed as follows:

1. The research team to conduct at least 90% of targeted household questionnaire interviews on day 1
2. At least 80% of consenting participants who completed the household questionnaire to go to the clinic on day 2 for clinical data collection
3. The selected clinic and skin prick test (SPT) procedures to comply with all the World Allergy Organization’s (WAO) safety recommendations for SPTs [28]
4. Prevalence of adverse/unintended reactions associated with SPTs to be less than 1%
5. Prevalence of mopane worm sensitisation to be at least 10% with or without suspected cross reactivity with other allergens in the panel
6. The mopane worm allergen would be deemed clinically relevant if at least one participant sensitised to it had lung function abnormalities and allergic airway inflammation suggestive of asthma

### Participant recruitment and data collection

The feasibility study was conducted in the village where the clinic selected for the study is located (Fig. 1). The village is relatively small with only 273 households and a total population of 1637 according to the District Health Information System (DHIS) records. Systematic random sampling was used to select households after limiting the study to a small geographic area, roughly within a 1-km radius around the clinic. To select the first household, we used the ‘spin-the-pen’ method that is detailed in the Expanded Program on Immunization (EPI) cluster survey design manual [29]. Using the clinic as the central location, a direction towards the households was selected randomly by spinning a pen. Moving in a straight line in that direction, 5 households along the pathway were counted and the third household was randomly selected as the first household. Thereafter, every second household was selected with the assistance of one of the local research assistants. Moving in a clockwise manner around the clinic, she guided the field team in finding an accessible path to the next eligible household. This was repeated until the desired number of participants was reached. Males and females aged 10 years and over up to a maximum of 4 participants (1 adult male, 1 adult female, 1 female child and 1 male child) per household, if available, were recruited. A total of 29 households were recruited. Written informed adult consent and, in cases of children between the ages of 10 and 17, parental consent and child’s assent were obtained. All consent forms were reviewed and received ethics approval.

### The main questionnaire

For data collection at the household, a comprehensive questionnaire whose questions were extracted from previously validated and standardised questionnaires was filled in by trained research assistants using Kobo Collect software [30]. The first section of the questionnaire had questions pertaining to demographic and socio-economic characteristics such as age, gender, education, occupation and monthly household income. The second section of the questionnaire interrogated knowledge, attitudes and practices with respect to asthma in the community. A series of dichotomous questions and Likert-scale-type questions on the knowledge of risk factors, signs, symptoms management and attitudes and perceptions for asthma were adapted from a previously validated instrument from the Chicago Community Asthma Survey (CCAS-32) [31]. The third and fourth sections of the questionnaire were, respectively, for the data collection of environmental exposure history (including residential history, occupational history and lifestyle factors such as smoking and alcohol consumption) and respiratory health questions including self-reported doctor-diagnosed asthma and asthma symptoms. To cater for the wide age range of the study population, relevant questions were extracted from the previously validated International Study of Asthma and Allergies in Childhood (ISAAC) [32] and The European Community Respiratory Health Survey questionnaire (ECRHS) [33] that has been used to collect information from adults. The feasibility outcome for the questionnaire was the total number that could be completed in 1 day against a set target of 46. Also important was to qualitatively assess the appropriateness of the questionnaire for the targeted audience. Therefore, at the end of each interview, participants were requested to comment on the clarity of questions, the length of the questionnaire or any other observation. After completing the main questionnaire, participants were invited to go to the clinic for further tests carried out in 3 key steps.

In step 1, a skin prick test was done to assess sensitisation to mopane worm and other locally relevant allergens included in the panel. The second step involved lung function assessment using spirometry and the third step was for FeNO tests to assess allergic airway inflammation. A qualified clinician was available on site in the event of any adverse reactions and arrangements were made at the nearby provincial hospital in the event that an emergency management became necessary.

### Assessment of allergen sensitisation to mopane worm and other important allergens

Demographic and clinical data including anthropometric data, cigarette smoking, history of alcohol consumption, history of influenza or sinusitis, tuberculosis, allergic rhinitis and dermatitis were collected using a clinic data collection sheet designed specifically for this study. Since spatial differences are found in the distribution and sensitisation patterns of allergens [7, 34], it was necessary to also include other allergen extracts in order to identify the most relevant panel for the study area. The choice of allergens to include was based on our prior knowledge of the environmental characteristics of Gwanda district and a previous study in another part of Zimbabwe that used a similar panel [13]. Their inclusion was also necessary in order to identify participants that are uniquely sensitised to mopane worm. Although mopane worm is an edible insect and would be considered a food allergen, the research focus was on its role as an inhalant allergen. Our motivation for this study was the excessive harvesting that is currently occurring which typically produces inhalable steam, aerosols or dust particles as a result of activities such as the cleaning, boiling and drying of mopane worm in preparation for sale [24, 35]. Respiratory allergy has been documented with increasing frequency in the food industry due to exposure to food allergens by inhalation [36, 37]. Allergen sensitisation patterns were, therefore, evaluated using 10 different inhalant allergen extracts. Participants were tested for allergen sensitisation to maize pollen, barley, 5 grass mix, cockroach, mosquito, the house dust mite (HDM) species *Dermatophagoides pteronyssinus* (D.pter.), *Dermatophagoides farinae* (D.far), and *Tyrophagus putrescentiae*, *Alternaria* and *Imbrasia belina* (mopane worm). Additionally, histamine (10 mg/ml) and saline (0.9% NaCl) were included in the panel as positive and negative controls respectively. The skin prick test extracts used were sourced commercially (Stallergenes, France) with the exception of mopane worm which was prepared in-house.

### Preparation of the mopane worm extract

Mopane worm allergy has only recently been recognised and there are currently no commercially available extracts for skin prick testing from any of the manufacturers. In order to perform SPT, a mopane worm saline extract was prepared at the Biochemistry Department of the University of Zimbabwe. The use of in-house extracts and prick to prick testing is standard practice in allergy testing [38, 39]. Established guidelines for extract preparation were followed [40, 41]. Dried mopane worms were purchased from a Zimbabwean supermarket. Worm extract was prepared by subjecting the worms to 3 cycles, a minute each, of alternate heating at 95 °C and freezing at − 195 °C in liquid nitrogen. Thereafter, 20 ml of Laemmli sodium dodecyl sulphate (SDS) sample buffer (Bio-Rad Laboratories, Hercules, CA, USA) [42] was added to 2.5 g of mopane worms and sonicated for 10 min using an Ultra Turrax (IKA Labortechnik, Staufen, Germany). This was followed by centrifugation at 14 000×*g* for 10 min. The supernatant was aspirated and stored at − 20 °C. Protein concentration was determined using spectrophotometry. The mopane worm in house skin prick test was prepared by diluting the extract to a concentration of 1.437 mg/dl using 0.9% sodium chloride.

### Interpretation of skin prick test results

Skin prick tests were performed at the clinic under the supervision of an allergy specialist. A drop of each test solution was placed on the volar aspect of the forearm at least 2 to 3 cm from the wrist. Each drop was immediately pricked with a sterile lancet and held against the skin for at least 1 s. Results were read 15–20 min following application [43]. Skin prick test wheal diameters exceeding 3 mm or greater than the saline control were considered as positive for sensitivity [44–46]. This definition of sensitisation has been used in several other studies in sub-Saharan Africa [2, 47, 48] including Zimbabwe [49, 50]. An observation checklist was used to rate the safety of the procedures against the WAO safety recommendations for SPTs [28].

### Lung function assessment

Lung function was assessed using a portable office spirometer (KoKo® Legend) in line with the American Thoracic Society (ATS) and European Respiratory Society (ERS) guidelines [51]. The spirometric parameters that were measured included forced vital capacity (FVC), forced expiratory volume in 1 s (FEV_1_), forced expiratory flow between 25 and 75% of FVC (FEF_25–75%_), the peak expiratory flow rate and FEV_1_/FVC ratio. A specialist nurse, specifically trained in spirometry, conducted all the spirometry tests after demonstrating the appropriate breathing manoeuvre and explaining it in vernacular language to the participants. Tests were performed while the participants were seated. At least 3 satisfactory measurements were done for each participant after which the best, according to the ATS/ERS guidelines, was selected for analysis [51, 52]. Lung function testing was discontinued if the participant was unable to produce acceptable results after 8 attempts. The spirometry equipment available to the research team at the time of data collection used the European Community for Coal and Steel (ECCS) reference values. Lung function parameters were, thus, expressed as a percentage of the predicted normal values according to the ECCS reference equation with a 10% adjustment for ethnicity [53].

### Fractional exhaled nitric oxide (FeNO) test to assess allergic airway inflammation

Fractional exhaled nitric oxide measurement is a recognised non-invasive method for assessing allergic airway inflammation [54, 55]. The FeNO test discriminates between different types of asthma and guides therapy. It is a novel way of confirming an asthma diagnosis and defining asthma theratypes. For participants with an abnormal measurement from at least one of the spirometric parameters, a fractional exhaled nitric oxide (FeNO) test to assess allergic airway inflammation was recommended. A hand-held portable nitric oxide sampling device (NIOX VERO® Airway Inflammation Monitor (NIOX VERO); Circassia, Oxford, UK) was used according to the manufacturer’s instructions and in line with the current American Thoracic Society/European Respiratory Society (ATS/ERS) recommendations [56]. A trained nurse carried out the tests to determine fractional exhaled NO (FeNO) from each eligible participant. The technique involved inspiration of NO-free air via a mouthpiece to total lung capacity, followed immediately by full exhalation at an even rate through the mouthpiece into the apparatus. The nurse carefully explained the technique, in vernacular, to the participants before carrying out the assessment. The ATS/ERS guidelines make evidence-based recommendations in the interpretation of FeNO levels and they have been used in African studies [57, 58]. Elevated FeNO levels greater than 50 ppb in adults and greater than 35 ppb in children indicate eosinophilic airway inflammation that is suggestive of probable allergic asthma. FeNO levels 25–50 ppm in adults and 20–35 ppb in children indicate possible airway inflammation but should be interpreted with caution and within the context of other clinical data collected such as the self-reported asthma symptoms and the skin prick test results [59].

### Data analysis

The software selected for data analysis was Stata Release 13 (StataCorp, TX, USA) [60]. Recruitment rate was calculated as a percentage of the target (46 participants) in 1 day. Participants’ comments after completing the questionnaire were reviewed qualitatively. Response rate for clinic data collection was calculated as a percentage of those who completed the questionnaire the day before. For normally distributed data, mean and standard deviation were used to summarise the variables. FeNO and spirometry variables were analysed after logarithmic transformation due to the skewness of the data and the results were presented as geometric means with 95% confidence intervals (CI). The *t* test for independent samples was used to compare mean values of log-transformed spirometry and FeNO measurements between the participants sensitised and those not sensitised to mopane worm extract. Categorical variables were summarised as frequencies and percentages. The Fisher’s exact test was used to detect differences in proportions of self-reported asthma and respiratory symptoms between participants sensitised to mopane worm and those who were not. This test was selected because of the small sample size and the expected frequency of less than 5 in each of the cells. A new discrete variable named ‘Polysensitisation’ was generated which indicated how many allergens each individual was sensitised to. Significance was considered for *p* values less than 0.05 for all the statistical tests performed.

## Results

**Fig. 2:**
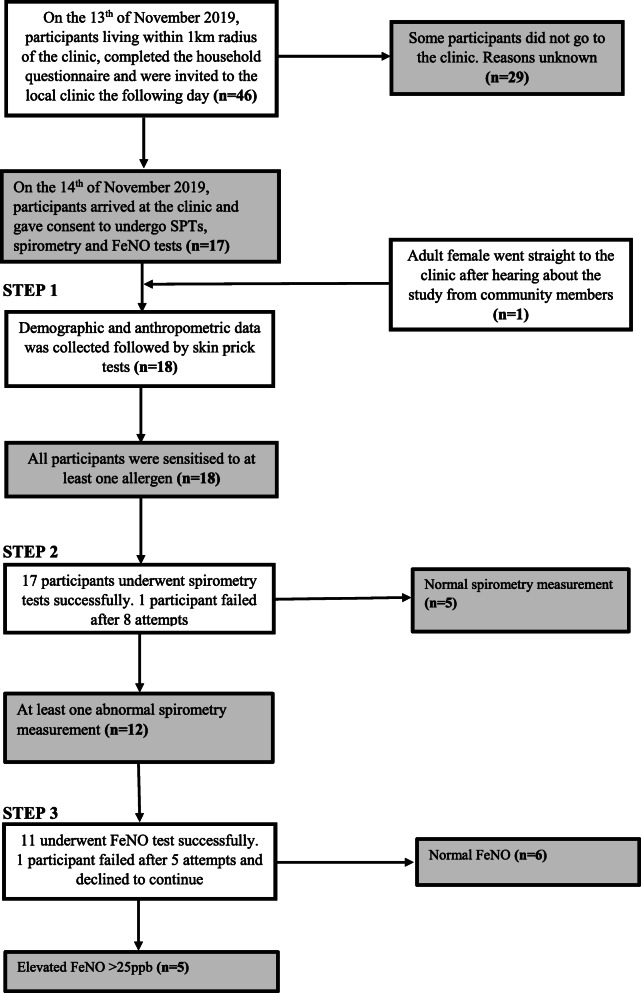
Flow diagram of the study procedure

The recruitment of participants and the data collection procedure is summarised in Fig. 2. On the 13th of November 2019, 46 eligible participants living within a 1-km radius of the clinic selected for this study completed the household questionnaire giving a recruitment rate of 100% against the set target. After completing the questionnaire, participants reported that it was relatively easy to answer largely because it was interviewer administered. However, for a number of dichotomous items (‘true/false’ or ‘yes/no’) testing knowledge, some participants indicated to the researchers that they did not know the answers to some of the questions and were forced to guess between the two available options. The consensus amongst participants was that the third section on environmental exposure history was too long. After completing the questionnaire, they were invited to the local clinic the following day and none of them declined the invite.

Part of the feasibility objectives for this study was to determine how many participants, following completion of the main questionnaire in their households, could attend the clinical assessments the following day. Out of the 46 participants that completed the questionnaire, a total of 17 eligible people went to the clinic on the day of data collection giving a lower than expected response rate of 37%. An 18th participant went straight to the clinic when she heard about the study but was lost to follow-up before completing the household questionnaire (Fig. 2). The field researchers were unable to locate her but were later informed that she left the village after receiving a job offer. She was a 43-year-old female with sensitisation to mopane worm and 4 other allergens. She could not be included in some of the analyses because of missing data. A summary of the participants’ demographic characteristics is presented in Table 1. Out of the 46 participants who completed the questionnaire, 91% were adults with a mean age of 47 ± 16.08 years (range = 18–72). The majority of participants were female with males only contributing 28% of the total sample population. The children in the study (9%) were all female with a mean age of 13 ± 2.71 years (range = 11–17).

**Table 1:** Comparison of demographic and clinical data between participants who completed the household questionnaire and the clinical assessments and those who completed the household questionnaire only

Variables	Participants’ data collection completion status		Total (***n*** = 46)
	Household questionnaire and clinic data collection completed (***n*** = 17)	Completed household questionnaire only (***n*** = 29)	
**Age (years), mean (± SD)**			
Children (*n* = 4)	11.5 (± 0.71)	14.5 (± 3.5)	13 (± 2.71)
Adults (*n* = 42)	49 (± 11.12)	46 (± 18.36)	47 (± 16.08)
**Gender,** ***n*** **(%)**			
Female	13 (76)	20 (69)	33 (72)
Male	4 (24)	9 (31)	13 (28)
**Education level,** ***n*** **(%)**			
No education	1 (5.88)	1 (3.45)	2 (4.35)
Primary	7 (41.18)	11 (37.93)	18 (39.13)
Secondary	5 (29.41)	17 (58.62)	22 (47.83)
Tertiary	4 (23.53)	0 (0)	4 (8.7)
**Marital status,** ***n*** **(%)**			
Married	12 (71)	22 (76)	34 (74)
Single	5 (29)	7 (24)	12 (26)
**Ever smoke,** ***n*** **(%)**	1 (6)	5 (17)	6 (13)
**Passive smoking,** ***n*** **(%)**	13 (76)	17 (59)	30 (65)
**Alcohol,** ***n*** **(%)**	0 (0)	5 (17)	5 (11)
**Tuberculosis,** ***n*** **(%)**	1 (6)	1 (3)	2 (4)
**Respiratory allergy symptoms on exposure to**			
Furry animals, *n* (%)	6 (35)	7 (24)	13 (28)
Trees, grass and other plants, *n* (%)	9 (53)*	6 (21)	15 (33)
**Harvest mopane worms,** ***n*** **(%)**	10 (59)	23 (79)	33 (72)
**Symptoms when harvesting mopane worm (*n*** **= 33),** ***n*** **(%)**	6 (60)	10 (43)	16 (48)
**Respiratory allergy outcomes,** ***n*** **(%)**			
Wheeze	9 (53)*	7 (24)	16 (35)
Woken up by chest tightness	6 (35)	6 (21)	12 (26)
Shortness of breath at rest	8 (47)*	4 (14)	12 (26)
Woken by cough	11 (65)*	10 (34)	21 (46)
Phlegm in the morning	9 (53)	15 (52)	24 (52)
Doctor-diagnosed asthma	2 (12)	1 (3)	3 (7)
Nasal allergies	4 (24)	4 (14)	8 (17)
Skin allergies	7 (41)	6 (21)	13 (28)

*Statistically significant differences in the proportion of these variables when comparing those who went to the clinic and those who did not (*p* < 0.05)

As a result of the low response rate on the second day, data was also analysed to compare participants who went to the clinic and those who did not on certain key demographic and clinic characteristics. No significant differences in demographic characteristics were found between those who completed clinic data collection and those who did not (Table 1). Differences were however found in self-reported respiratory symptoms. The participants who went to the clinic had reported higher proportions of some respiratory health symptoms such as wheeze and cough in the last 12 months.

The WAO safety recommendations were used to assess the safety of the SPT procedures in the selected clinic (Table 2). It was concluded that it was possible to maintain the safety standards required to successfully conduct the tests. Furthermore, there were no recorded adverse events.

**Table 2:** Compliance to the WAO safety recommendations for skin prick testing with inhalant and food allergens

Criteria	Safety recommendation	Procedure during the pilot	Conclusion on compliance and feasibility status
Site	A hospital and outpatient clinic setting	Fully equipped local clinic with four qualified nurses	Compliant and feasible
Personnel	SPTs can be performed by a trained nurse/technician under the supervision of an experienced physician	Three trained local nurses as well as a laboratory technician and specialist nurse both working in an allergy clinic performed the SPTs under the supervision of an allergy specialist	Compliant and feasible
Emergency equipment availability	Should be available on site (mandatory)	An allergy emergency kit was available at the fully functional clinic with basic emergency equipment and medication	Compliant and feasible
Emergency staff (ICU) availability	Not required	Arrangements were made with the provincial hospital to be on standby in the event of an adverse event	Compliant and feasible
Duration of supervised follow-up in the office after procedure	Participants who have undergone SPTs and have positive results should remain in the clinic for at least 20 min	Participants remained for at least 20 min and were informed to return immediately if there were problems	Compliant and feasible
Contraindications	Knowledge of contraindications in order to take appropriate action	Demographic, anthropometric and medical history data was collected followed by skin prick tests	Compliant and feasible

**Fig. 3:**
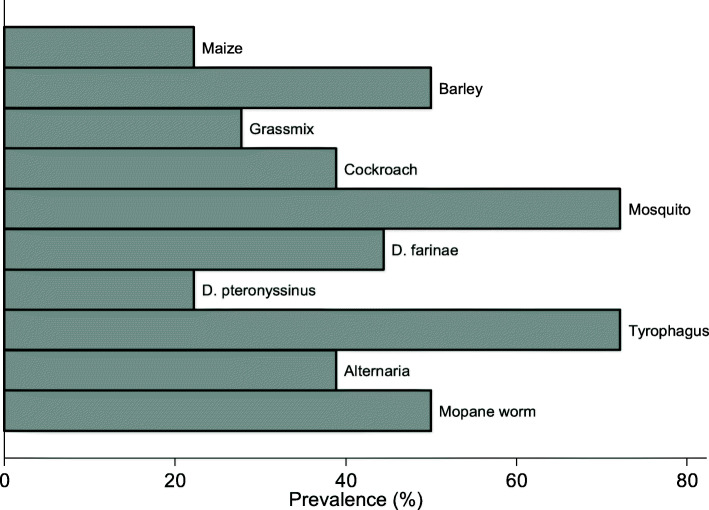
Prevalence of sensitisation to mopane worm and other allergens selected for the study

The prevalence of sensitisation to each of the allergens under study is presented in Fig. 3. Allergen sensitisation was more common than expected in this rural community. Half of the participants were sensitised to *Imbrasia belina*.

**Fig. 4:**
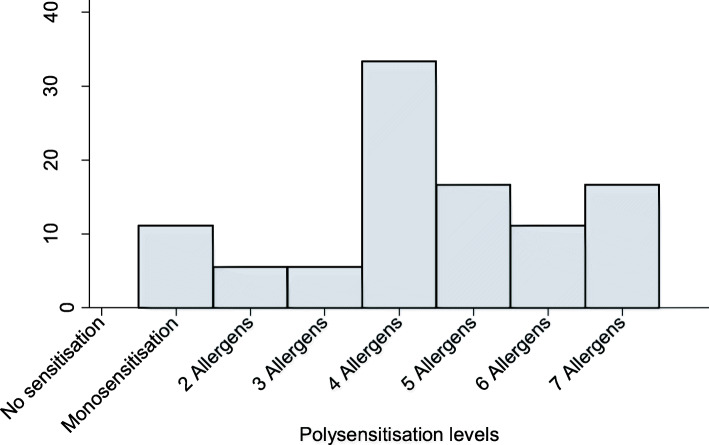
Sensitisation patterns indicating the distribution of polysensitisation to allergens

The most frequent sensitisers were mosquito (72%) and *Tyrophagus* (72%) and the least frequent were maize (22%) and the house dust mite species *Dermatophagoides pteronyssinus* (22%). Additionally, Fig. 4 shows that all participants were sensitised to at least one allergen and polysensitisation, defined as sensitisation to two or more allergens [61], was common. Monosensitisation was observed in two of the participants whereby one was sensitised to mosquito and the other was sensitised to the house dust mite *Dermatophagoides farinae*. A third of the participants (33%) were sensitised to 4 allergens.

**Fig. 5:**
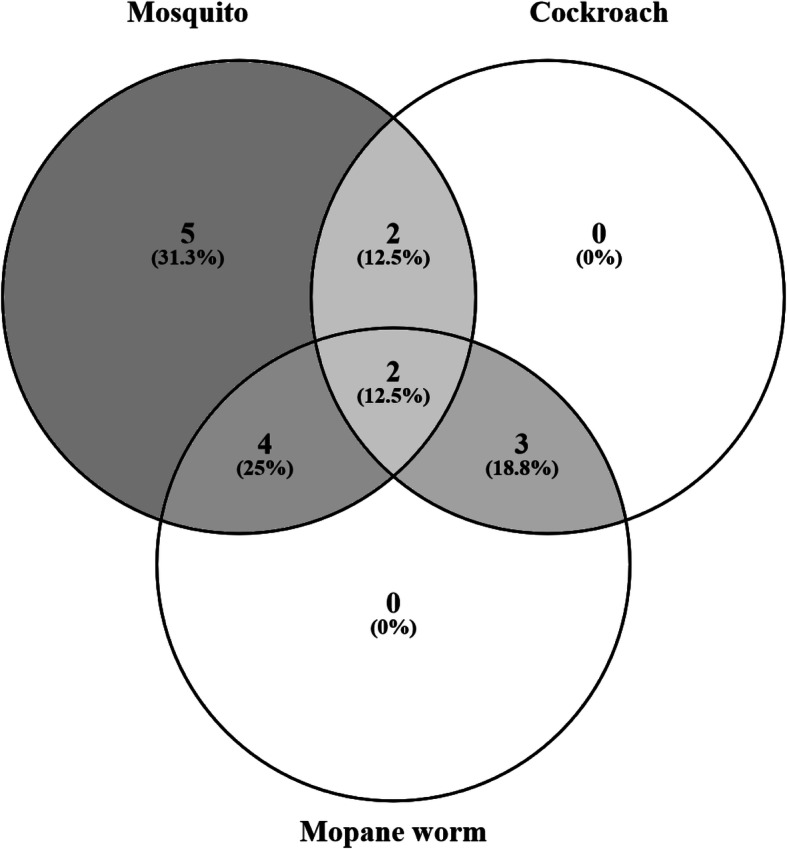
A Venn diagram illustrating co-sensitivity or possible cross reactivity between mosquito, cockroach and mopane worm allergens

A Venn diagram was generated [62] to look for evidence of co-sensitisation or possible cross reactivity amongst three allergens belonging to the same taxonomic class *Insecta* namely cockroach, mosquito and *Imbrasia belina*. None of the participants was uniquely sensitised to mopane worm as shown in Fig. 5. Those with mopane worm sensitisation either had mosquito or cockroach sensitisation or both.

In addition to quantifying the prevalence of sensitisation to mopane worm, we assessed whether the sensitisation was clinically relevant. Comparison of self-reported history of mopane worm harvesting and respiratory health symptoms by mopane worm sensitisation status is summarised in Table 3. Fifty-nine percent of the participants reported a history of mopane worm harvesting. There were similar proportions of harvesters in the sensitised and the non-sensitised groups. Furthermore, amongst those sensitised to mopane worm, half were not harvesters.

**Table 3:** Comparison of self-reported history of mopane worm harvesting and respiratory health symptoms by mopane worm sensitisation status

Outcome variables	Prevalence, *n* (%)		
	Sensitised to mopane worm (***n*** = 8)	Not sensitised to mopane worm (***n*** = 9)^a^	Total (***n*** = 17)
**History of mopane worm harvesting**			
Harvest mopane worms	4 (50)	6 (67)	10 (59)
Duration of harvesting in years, mean (± SD)	38 (± 9.8)	15.4 (± 13.8)	25 (± 16.5)
Symptoms when harvesting mopane worm (*n* = 10)	2 (50)	4 (67)	6 (60)
**Respiratory health symptoms**			
Wheeze*	4 (50)	5 (56)	9 (53)
Woken up by chest tightness	2 (25)	4 (44)	6 (35)
Woken by shortness of breath	3 (38)	4 (44)	7 (41)
Shortness of breath at rest	4 (50)	4 (44)	8 (47)
Shortness of breath following exercise	3 (38)	5 (56)	8 (47)
Woken by cough	5 (63)	6 (67)	11 (65)
Morning phlegm	4 (50)	5 (56)	9 (53)
Doctor-diagnosed asthma	1 (13)	1 (11)	2 (11)

*Wheeze came with breathlessness and was present even in the absence of a cold

^a^No significant differences between the two sensitisation groups using Fisher’s exact test

Responses to the questionnaire on asthma and respiratory symptoms in the last 12 months are presented in Table 3. The most frequently reported symptom was cough (65%) followed by wheeze (53%) and morning phlegm (53%). Participants that reported wheeze also indicated that the wheezing came with breathlessness and was present even in the absence of a cold. Participants sensitised to mopane worm and those who were not sensitised were compared to determine if there were differences in the proportions of self-reported asthma and respiratory symptoms (Table 3). There were no significant differences between the two groups.

There were no significant differences in lung function and allergic airway inflammation between those sensitised and those who were not sensitised to mopane worms (Table 4). Considering the small sample size, however, there was a substantial number of participants with abnormal predicted FVC (53%) and FEV_1_ (40%) as shown in Table 4. There were two participants (one male adult and one female adult) in this study that reported they had doctor-diagnosed asthma. Both had abnormal spirometric parameters and elevated FeNO in addition to being sensitised to 7 allergens each.

**Table 4:** Lung function and allergic airway inflammation

Outcome variables	Sensitised to mopane worm (*n* = 9)	Not sensitised to mopane worm (*n* = 8)	Total (*n* = 17)
**Geometric mean (95% CI)**^**b**^			
FVC (L)	2.95 (2.33–3.75)	2.35 (2.02–2.75)	2.65 (2.3–3.06)
FVC % predicted	81.23 (67.63–97.56)	75.39 (65.2–87.18)	78.06 (70.55–86.38)
FEV_1_ (L)	2.46 (1.88–3.22)	2.08 (1.63–2.66)	2.27 (1.92–2.69)
FEV_1_ % predicted	84.37 (67.99–104.71)	83.4 (68.68–101.29)	83.85 (74.05–94.96)
FEF_25–75%_ (L/s)	2.62 (1.79–3.85)	2.49 (1.55–3.98)	2.56 (1.96–3.33)
FEF_25–75 %_ predicted	101.36 (54.47–188.62)	90.82 (59.72–138.13)	95.95 (69.14–133.15)
FEV_1_/FVC	0.85 (0.80–0.90)	0.88 (0.78–0.98)	0.86 (0.82–0.91)
FEV_1_/FVC % predicted	95.73 (84.34–108.66)	110.58 (98.41–124.26)	102.45 (94.07–111.59)
FeNO (ppb)	16.4 (5.25–51.23)	26.23 (8.03–85.69)	21.19 (10.7–41.94)
***n*** **(%)**^**c**^			
Elevated FeNO (> 25 ppb) (total *n* = 11)	2 (40)	3 (50)	5 (45)
Abnormal FVC (< 80% Pred) (total *n* = 15)	3 (43)	5 (63)	8 (53)
Abnormal FEV_1_ (< 80% Pred) (total *n* = 15)	4 (57)	2 (25)	6 (40)
Abnormal FEF_25–75%_ (< 80% Pred) (total *n* = 16)	3 (38)	2 (25)	5 (31)

*FEV*_*1*_ forced expiratory volume in 1 s, *FVC* forced vital capacity, *FEF*_*25–75%*_ forced expiratory flow at 25 to 75% of forced vital capacity, *FENO* fractional exhaled nitric oxide

^b^No differences between the two sensitisation groups using the two-sample Wilcoxon rank-sum (Mann-Whitney) test

^c^No significant differences between the two sensitisation groups using Fisher’s exact test

## Discussion

Prior to conducting the GARAS study in Garanyemba, a rural area in Zimbabwe, we needed to demonstrate the feasibility of the proposed methodology by addressing key uncertainties. Apart from the limited budget, we were uncertain about the recruitment of eligible participants, response rates on invitation to the clinic, safety and acceptability of the data collection tools, plausibility of population-wide sensitisation to mopane worm and possible clinical relevance. Feasibility criteria were established a priori to address the concerns and make an appropriate decision about the main study. This study was able to meet all the pre-specified feasibility criteria with the exception of the lower than expected response rate for clinic data collection.

After the lower than anticipated response rate at the clinic, data was analysed further to determine if there were any systematic differences between those who decided to go the clinic and those who did not. Participants who went to the clinic had reported higher proportions of respiratory health symptoms such as wheeze and cough in the last 12 months than those who did not go to the clinic. This indicates that they may have been systematically more motivated to go the clinic as a result of the respiratory symptoms thereby introducing selection bias and likely overestimating the true prevalence of sensitisation to mopane worm and other outcomes of interest in this study. Even though the intention was to recruit a random sample of households, we recognise that it might not be feasible for the main study in light of the very low response rate at the clinic. The effect of distance was eliminated in this study by recruiting individuals close to the clinic. We thus believe that fear, mistrust and uncertainty about the clinic data collection procedure may have led to the low response rate, a common phenomenon in conservative communities [63, 64]. The decision to collect clinic data on day 2, though deemed necessary, also contributed towards the low response rate. Taking into consideration that participants residing even further away from the clinic than a radius of 1 km will be recruited during the main study, it has been deemed necessary to modify the current sampling strategy to a nonprobability method. To minimise non-response for the clinical data collection phase during the main study, we will use the volunteer sampling method to recruit participants who are willing to come to the clinic. Additionally, the willing participants will complete the questionnaire at the clinic and have the clinical assessment done on the same day. Transport costs will be reimbursed. A study awareness campaign will be conducted, in all the 8 villages of the ward, with the assistance of the community leaders in order to build trust between researchers and the community. Adult males and children were underrepresented in the study because the study was conducted during the week while children were at school and men were at work. To improve response rates from both population groups during the main study, data will also be collected during weekends and every effort will be made for fieldwork to coincide with the school holiday. There will also be more training and coordination between the clinic team and field team to ensure that no participants are lost to follow-up before data collection is complete.

The data collection tools were safe and well tolerated by participants with no adverse events reported. The study also demonstrated that it was feasible to comply with the WAO safety recommendations in the selected clinic. The selected devices for spirometry and FeNO were portable and had rechargeable batteries. They were convenient to use in remote settings and offered reliable readings.

One of the main objectives of this pilot study was to assess the plausibility of widespread mopane worm sensitisation in Gwanda district, a mopane worm harvesting rural community. We determined that it was plausible as 50% of the participants were sensitised to mopane worm. In this study, being a mopane worm harvester did not appear to be the only determinant for mopane worm sensitisation as there were approximately equal proportions of harvesters in both the sensitised and the non-sensitised groups. Furthermore, amongst those sensitised to mopane worm, 50% were not harvesters. A limitation in this pilot study is that we did not examine various other opportunities for exposure to mopane worm other than harvesting. Moreover, harvesting is an elaborate process that typically involves hand picking from the mopane trees, degutting, boiling and drying [35]. Thereafter, there could be selling and/or eating of the mopane worms. All these are possible ‘exposures’ to mopane worm that could offer explanations for sensitisation because allergens can enter the organism mostly by inhalation, ingestion or after skin contact [65, 66]. We therefore intend to factor in more ‘exposure’ variables in the main study as well as to identify host factors influencing susceptibility to mopane worm sensitisation. Though the inclusion criteria in this pilot study were males and females aged 10 years and above, the study population was dominated by adult females. The sample size was too small to make meaningful comparisons of sensitisation by gender or by age.

Knowledge about local sensitising allergens, the sensitisation patterns and their degree of exposure in the community is essential because it aids the diagnosis and subsequent treatment of allergic respiratory diseases [67]. The results showed that polysensitisation was surprisingly very common in this rural community. Because the presence of polysensitisation occurs as a result of either cross reactivity or co-sensitisation [61], it was difficult to establish whether a positive mopane worm skin prick test was due to true sensitisation or cross-reactivity. To explore this, we constructed a Venn diagram including mopane worm, mosquito and cockroach all belonging to the *Insecta* class. None of the participants appeared uniquely sensitised to mopane worm; therefore, a future study is recommended to identify and characterise the allergens that are specific to the worm.

Even more important than assessing the prevalence of mopane worm sensitisation was to determine whether it was a clinically relevant allergen and therefore of public health importance. The mopane worm allergen was able to meet the pre-specified feasibility criterion of clinical relevance whereby at least one participant sensitised to it also had an abnormal lung function and allergic airway inflammation suggestive of asthma. Abnormal spirometry and elevated FeNO were statistically not associated with mopane worm sensitisation. This was expected because of the small sample size and precludes any conclusions being drawn from this observation. These variables will be further explored in the main study with sufficient sample size. Moreover, there was a sizeable number of participants that had abnormal spirometry measurements. It might be sensible to explore other chronic lung diseases for future research because the study area is in the region that has the highest prevalence of HIV in the country [68, 69]. HIV-infected populations have been found to have a higher risk of chronic lung diseases [70, 71].

We believe a large part of the success in achieving most of the feasibility criteria in this study was attributed to the community engagement activities that took place prior to the study. While we cannot conclude that the findings are generalisable to similar settings, we hope our experiences could be invaluable to other researchers considering the feasibility of conducting asthma studies utilising objective data collection tools in conservative and remote African communities.

## Conclusions

This pilot study has demonstrated that our protocol, with the exception of the sampling strategy, successfully met the pre-defined feasibility criteria. In conclusion, the pilot study provides useful insights about the underlying sensitisation patterns in the rural community of Gwanda district in Zimbabwe. Sensitisation was common, especially to insects. Whether there is any significant relationship between sensitisation to local allergens and respiratory allergy in this community requires further investigation in the main study with sufficient sample size.

## Supplementary Information

**Additional file 1.** CONSORT Extension Pilot and Feasibility Trials Checklist

## Data Availability

The datasets used and/or analysed during the current study are available from the corresponding author on reasonable request.

## Abbreviations

GARAS: Gwanda Asthma and Respiratory Allergy Study
CE: Community engagement
WAO: World Allergy Organization
SPT: Skin prick test
CCAS-32: Chicago Community Asthma Survey
ATS: American Thoracic Society
ERS: European Respiratory Society
FVC: Forced vital capacity
FEV_1_: Forced expiratory volume in 1 s
FEF_25–75%_: Forced expiratory flow between 25 and 75% of FVC
ISAAC: International Study of Asthma and Allergies in Childhood
ECRHS: European Community Respiratory Health Survey
ECCS: European Community for Coal and Steel
WHO: World Health Organization
STROBE: Strengthening the Reporting of Observational Studies in Epidemiology checklist
FeNO: Fractional exhaled nitric oxide
